# Role of m6A modification and novel circ_0066715/ miR-486-5p/ ETS1 axis in rheumatoid arthritis macrophage polarization progression

**DOI:** 10.18632/aging.204439

**Published:** 2022-12-20

**Authors:** Lei Wan, Jian Liu, Chuanbing Huang, Ziheng Zhu, Fangze Li, Guanghan Sun, Kun Wang, Shu Li, Ximeng Ma, Xi Chen, Wang Yuan

**Affiliations:** 1 The First Affiliated Hospital of Anhui University of Chinese Medicine, Hefei 230038, China; 2 Key Laboratory of Xin’an Medical Education Ministry, Hefei 230038, China; 3 College of Traditional Chinese Medicine, Anhui University of Chinese Medicine, Hefei 230012, China

**Keywords:** N6-methyladenosine methylation modification, circular RNA, microRNA, rheumatoid arthritis, macrophage polarization

## Abstract

Rheumatoid arthritis (RA) is a systemic disease dominated by inflammatory synovitis. RA synovial macrophages tend undergo M1-type macrophage polarization. Then, polarized M1-type macrophages secrete abundant pro-inflammatory cytokines, causing joint and cartilage destruction. N6-methyladenosine (m6A) methylation modification, circular RNA (circRNA), microRNA (miRNA), messenger RNA (mRNA), etc. are involved in the inflammatory response of RA. We found that there is an imbalance of inflammatory polarization in RA, which is manifested by a sharp increase in inflammatory markers and a high inflammatory response. Here, we show that RA was closely associated with low expression of circ_0066715. The overexpression of circ_0066715 significantly increased the ETS1 levels in RA-FLS cells, decreased cytokine secretion by M1-type macrophages, elevated M2-type cytokines, and inhibited FLS proliferation. Interestingly, the overexpression of miR-486-5p significantly suppressed the attenuation of the cell function and the effect on M1 macrophage polarization caused by circ_0066715 positive expression. WTAP may be involved in the methylation process of ETS1 in RA. ETS1 m6A methylation levels were altered upon WTAP intervention. The overexpression or interference of circ_0066715 decreased or increased WTAP expression. Our findings provide a novel circRNA/miRNA/mRNA regulatory axis and m6A regulatory mechanism involved in the process of RA macrophage polarization, thereby providing a powerful diagnostic and therapeutic strategy for RA treatment.

## INTRODUCTION

Rheumatoid arthritis (RA) is a chronic inflammatory disease. The main features of RA are joint synovial inflammation and infiltration of innate and adaptive immune cells [1, 2]. In RA, a large number of immune cells such as macrophages and lymphocytes are accumulated in the two layers of the synovial tissue [3, 4]. Macrophages and lymphocytes overproduce pro-inflammatory mediators, including TNF-α, IFN-γ, and IL-6., which promotes bone erosion and adjacent cartilage and bone destruction [5]. Macrophages are involved in multiple aspects of the inflammatory response in RA, such as neovascularization stimulation and the recruitment of neutrophils, monocytes, and lymphocytes. Furthermore, fibroblast proliferation and protease secretion are promoted. These processes eventually lead to joint destruction [6–8]. In addition, peripheral blood and synovial and synovial fluid macrophages in RA patients tend to polarize toward M1 macrophages [9, 10]. Next, polarized M1 macrophages secrete a large number of pro-inflammatory cytokines (TNF-α, IL-6, IL-12, iNOS, etc.), as well as various matrix enzymes. Fibroblasts and osteoclasts are then activated, and neutrophils, monocytes and lymphocytes are recruited, triggering a series of inflammatory responses. That accelerated bodily inflammation causes joint/ cartilage damage [11–13].

Previous Studies have showed that N6-methyladenosine (m6A) methylation modification, circular RNA (circRNA), microRNA (miRNA), messenger RNA (mRNA), etc., play an important role in the occurrence and development of RA [14, 15]. Studies have shown that N6-methyladenosine (m6A) RNA modification on human circRNAs inhibits innate immunity [16]. Claudio’s findings show that the m6A mark acts as a key post-transcriptional modification that promotes the initiation of miRNA biogenesis [17]. Meanwhile, their effects are closely related to RA disease activity. They are also involved in the pathological process of RA pathogenesis, including immune inflammation and escape from apoptosis. The study showed that the parental genes of m6A-modified transcripts were selectively enriched in multiple signaling pathways including PI3K-Akt, MAPK, JAK-STAT, and Wnt signaling pathways. These pathways are interconnected and can be together involved in the processes of RA such as macrophage polarization, inflammatory response, and synovial fibroblast proliferation [18, 19]. However, the underlying mechanisms of m6A methylation modification and circRNA, miRNA, and mRNA participation in RA immune inflammatory response have not yet been elucidated. In this paper, we further investigated ceRNA regulation of macrophage polarization in RA, which leads to the occurrence of RA immune inflammation.

## RESULTS

### Expression of M1 and M2 macrophages in RA patients

In RA, the expression of M1 macrophage marker CD14+CD86+ was higher than that in the HC group, whereas the expression level of M2 macrophage marker CD14+CD163+ was lower. Additionally, we found that the CD86+/CD163+ expression in the RA group was higher than that in the HC group (Supplementary Figure 1A–1E). These results suggested the presence of an M1/M2 expression imbalance in RA patients. Further observation showed that the expression levels of the cytokines IL-1R1, IFN-γ, and iNOS2 secreted by M1 macrophages in the RA group were significantly higher than those in the HC group. Conversely, the levels of the cytokines MRC1 and IL-10 secreted by M2-type macrophages were decreased (Supplementary Figure 1F–1J), which indicates an imbalance of inflammatory polarization in RA patients.

### Correlation analysis of macrophages and clinical indicators in RA

Correlation analysis showed that CD14+CD86+ in RA patients was positively correlated with CRP, ESR, RF, and DAS-28. However, CD14+CD163+ was negatively correlated with ESR and DAS-28 but positively correlated with CRP, ESR, RF, and CCP. The cytokines IL-1R1 and iNOS2 secreted by M1 macrophages were positively correlated with RA-related indicators, such as RF, CCP, CRP, ESR, and DAS28. IFN-γ was positively correlated with CCP, CRP, and ESR. The cytokine MRC1 secreted by M2 macrophages was negatively correlated with RF, CCP, CRP, ESR, and DAS28. Additionally, IL-10 was negatively correlated with CCP, CRP, and ESR (Supplementary Figure 2A–2G). These data suggest that there was an inflammatory polarization imbalance in RA, which was manifested by a sharp increase in inflammatory indicators and a strong inflammatory response.

### Screening of circ_0066715 and downregulation in RA

**Figure 1:**
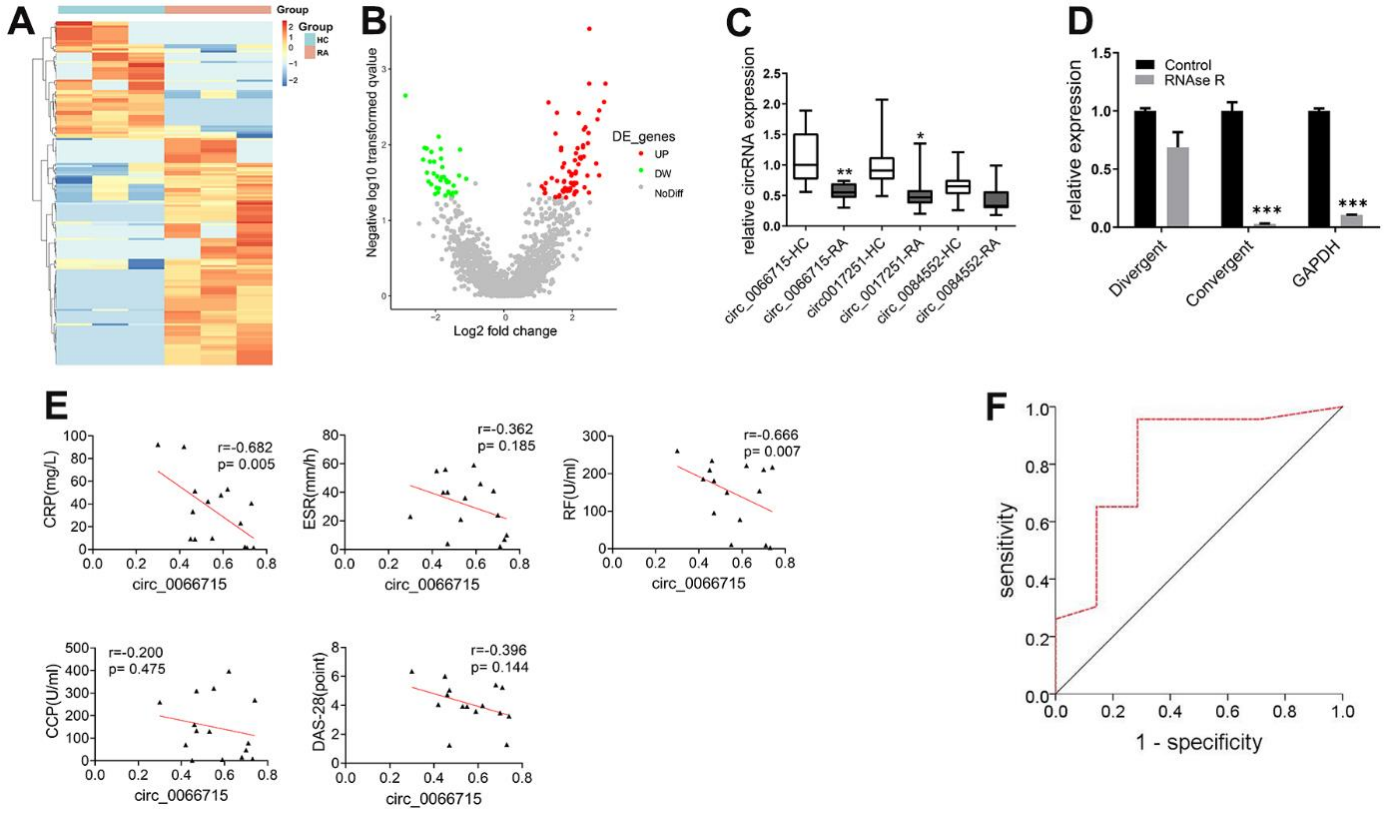
**Screening of circ_0066715 and its role in RA.** (**A**) Heat map of circRNA differential expression in PBMC of RA patients. (n=3). (**B**) Volcano plot of circRNA differential expression in PBMC of RA patients. (n=3). (**C**) Comparison of differential expression of three circRNAs in RA patients. **P<0.01, vs circ_0066715-HC. *P<0.05, vs circ_0017251-HC. (n=15, paired t-test). (**D**) Circ_0066715 feature verification in RA-FLS cells. ***P < 0.001,**P < 0.01,*P < 0.05.(n=3, paired t-test). (**E**) Correlation analysis between circ_0066715 and clinical indicators of in RA patients. (n=15, Spearman rank correlation analysis). (**F**) ROC curve of circ_0066715 and disease in RA patients. AUC=0.823, *P=*0.011, 95% CI: 0.**P < 0.01623~1.000.(n=30, ROC curve analysis).

High-throughput gene sequencing was used to screen differentially expressed circRNAs based on peripheral blood PBMCs of RA patients. A total number of 64 differentially expressed circRNAs were found by circRNAs gene sequencing analysis (difference fold ≥ 2, and *P* ≤ 0.05). Of them, 49 were upregulated, whereas 15 were downregulated. The 10 most significantly upregulated and downregulated circRNAs and mRNAs were clustered and analyzed. The results showed consistent gene expression trends among the samples (Figure 1A, 1B). The high-throughput sequencing results and circRNA sequencing GEO data showed that three circRNAs were differentially expressed (circ_0066715, circ_0017251, and circ_0084552). We then collected (15/30) matched RA and HC blood samples to establish their expression levels. qRT-PCR results revealed that only circ_0066715 was significantly lower in RA than in HC samples (Figure 1C). Due to the relatively large differential expression of circ_0066715, we selected circ_0066715 for follow-up studies.

To test the characteristics of circ_0066715, divergent primers and convergent primers were respectively designed, and cDNA and gDNA were used as templates for amplification detection. The linear circ_0066715 was almost completely digested by RNase R. However, circ_0066715 remained unchanged in the synovial cells. Next, we aimed to confirm the presence of circular RNA and found that circ_0066715 was a true circRNA (Figure 1D). Further observation of the relationship between circ_0066715 and RA inflammation was performed. circ_0066715 was inversely proportional to CRP and RF (Figure 1E). In addition, the ROC curves showed that RA patients with low levels of circ_0066715 had higher disease activity than those with high levels of circ_0066715. Therefore, RA disease activity was closely related to the low expression of circ_0066715 (Figure 1F). The above results indicate a nice correlation between cir_006675 and RA clinical markers. Meanwhile, along with the elevation of cir_006675, the inflammatory factors in RA patients also increased, further aggravating the RA disease progression.

### High circ_0066715 expression inhibits the proliferation of RA-FLS cells

**Figure 2:**
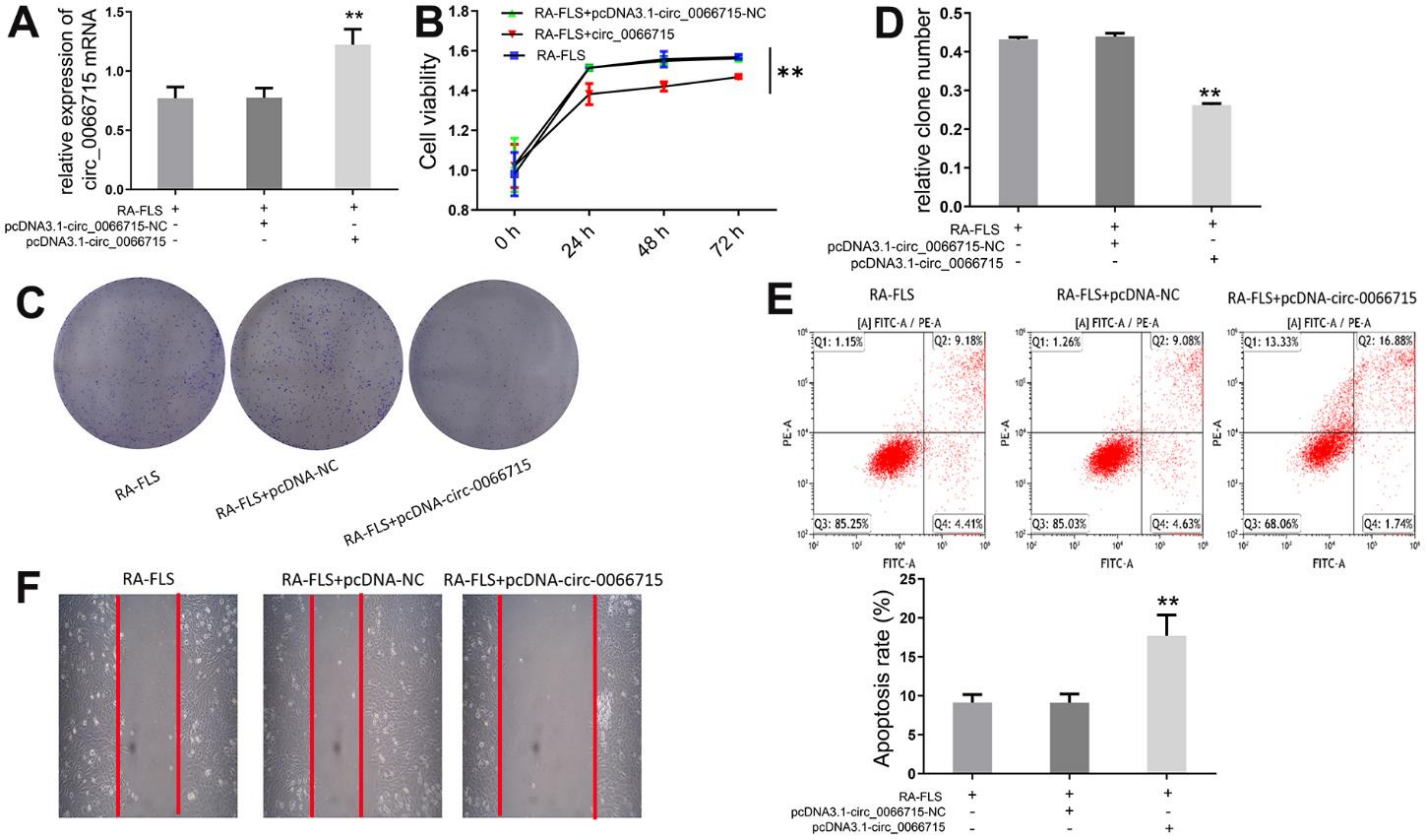
**Effect of circ_0066715 on the function of RA-FLS cells.** (**A**) circ_0066715 lentiviral vector construction in RA-FLS cells.** *P* <0.01.(n=6, ANOVA with Bonferroni’s post test). (**B**) Changes of cell viability. ** *P* <0.01.(n=3, ANOVA with Bonferroni’s post test). (**C**, **D**) Changes in cell clones.** *P* <0.01.(n=6, ANOVA with Bonferroni’s post test). (**E**) Changes of apoptotic. (**F**) Changes of cell scratch.

Next, we investigated the biological function of circ_0066715. A RA-FLS cell line stably expressing circ_0066715 was constructed using the aforementioned overexpression vector. The expression of circ_0066715 was significantly increased in the circ_0066715-overexpressing RA-FLS cell line (Figure 2A). CCK-8 analysis showed that RA-FLS cell viability was considerably attenuated after circ_0066715 overexpression (Figure 2B). Notably, fewer clones were formed in circ_0066715 overexpression wells compared with control wells (Figure 2C, 2D). Moreover, scratch experiments revealed that the overexpression of circ_0066715 inhibited RA-FLS cell migration and promoted repair (Figure 2E, 2F). These results indicated that circ_0066715 inhibits the proliferation and apoptosis of RA-FLS cells.

### circ_0066715 exerts a miRNA sponge-like effect of miR-486-5p

**Figure 3:**
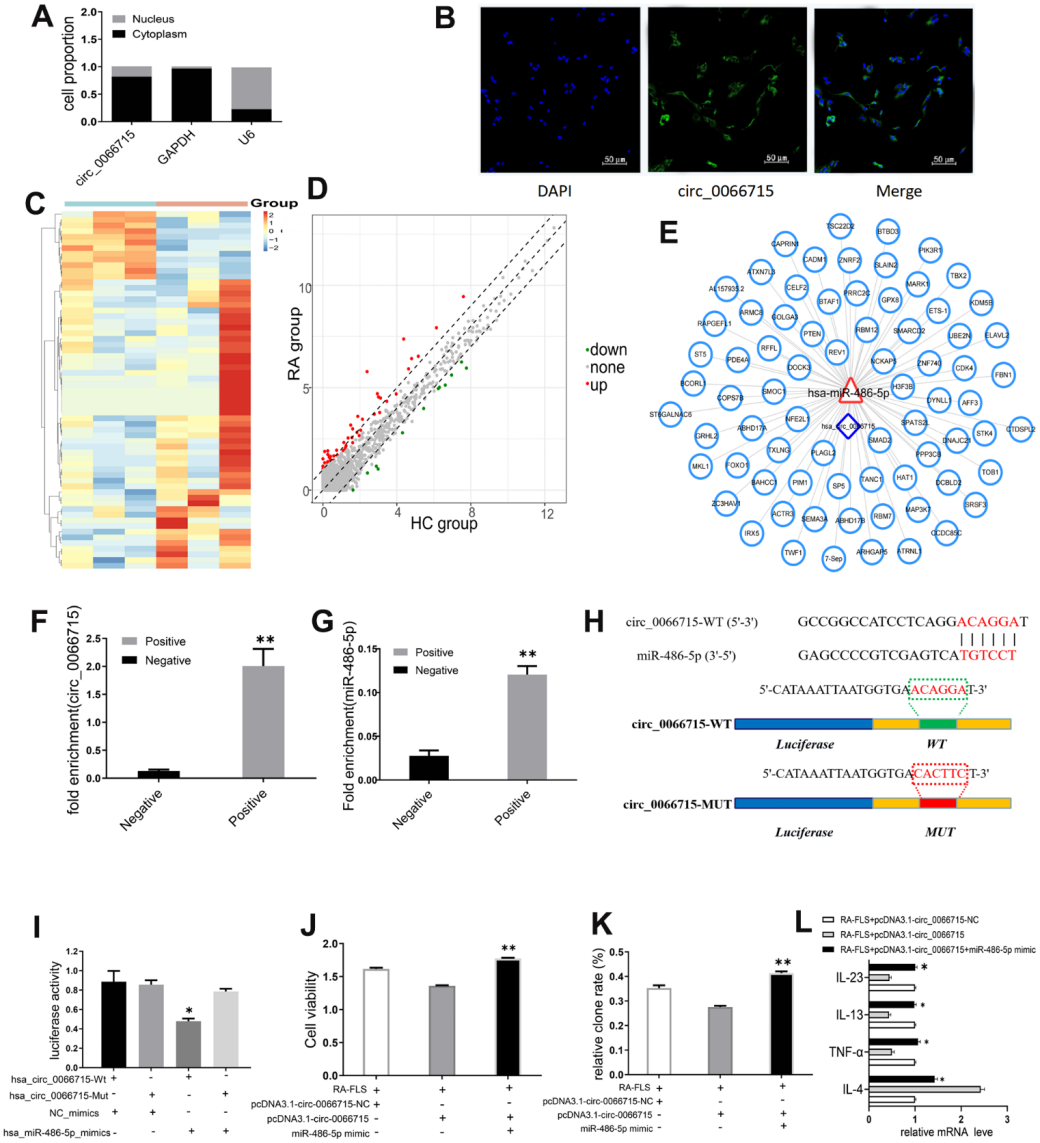
**circ_0066715 acts as a miRNA sponge-like effect of miR-486-5p.** (**A**) Nucleocytoplasmic separation experiments in RA-FLS cells. (n=9). (**B**) circ_0066715 localization in RA-FLS cells.(IF,×200). (**C**) Heatmap of differentially expressed miRNAs in PBMC of RA patients. (n=3). (**D**) Volcano plot of differentially expressed miRNAs in PBMC of RA patients. (n=3). (**E**) Circ_0066715 binding map with miRNA targeting. (**F**) RNA_ pull-down assay enrichment result of circ_0066715.**P < 0.01, vs Negative. (n=3, paired t-test). (**G**). RNA pull-down assay enriched miR-486-5p results. **P < 0.01, vs Negative. (n=3, paired t-test). (**H**) Binding site between miR-486-5p and circ_0066715. (**I**) Luciferase reporter assay. * *P* < 0.05, vs hsa_circ_0066715-Mut. (n=3, paired t-test). (**J**) Analysis of cell viability after targeting miR-486-5p with circ_0066715.**P < 0.01. (n=3, ANOVA with Bonferroni’s post test). (**K**) Clonal analysis of cells after targeting miR-486-5p with circ_0066715. **P < 0.01. (n=3, ANOVA with Bonferroni’s post test). (**L**) Cytokine expression after circ_0066715 targeted miR-486-5p in RA patients. * *P* < 0.05, vs RA-FLS+pcDNA3.1-circ_0066715. (n=15, paired t-test).

qRT-PCR and FISH analysis showed that circ_0066715 was located in the RA-FLS cytoplasm (Figure 3A, 3B), implying that it may act through spongy miRNAs. Through high-throughput sequencing, we found 33 differentially expressed miRNAs in the samples of the RA patients (difference fold ≥2, and *P* ≤ 0.05); 26 of them were upregulated, whereas 7 were downregulated (Figure 3C, 3D). The binding potential of circ_0066715 to miRNA targeting was predicted by RNAhybRid (Figure 3E). Circ_0066715 was found to have potential sites for binding to miR-486-5p. To verify the predicted results, we performed RNA pull-down assay and established that only the circ_0066715 probe in RA-FLS cells was enriched for miR-486-5p (Figure 3F, 3G). We mutated it and performed luciferase reporter gene analysis. The results showed that the overexpression of miR-486-5p caused a significant decrease in the luciferase activity of the wild-type circ_0066715 vector without affecting the mutant (Figure 3H, 3I). Functionally, in RA-FLS cells, miR-486-5p overexpression significantly blocked the attenuated cell viability and cell clone numbers caused by the positive expression of circ_0066715 (Figure 3J, 3K). Meanwhile, the overexpression of circ_0066715 significantly increased the IL-4 levels but decreased those of TNF-α, IL-13, and IL-23 levels in the RA-FLS. The overexpression of miR-486-5p effectively abolished the aforementioned effects (Figure 3L).

### circ_0066715 regulates the miR-486-5p/ETS1 axis

**Figure 4:**
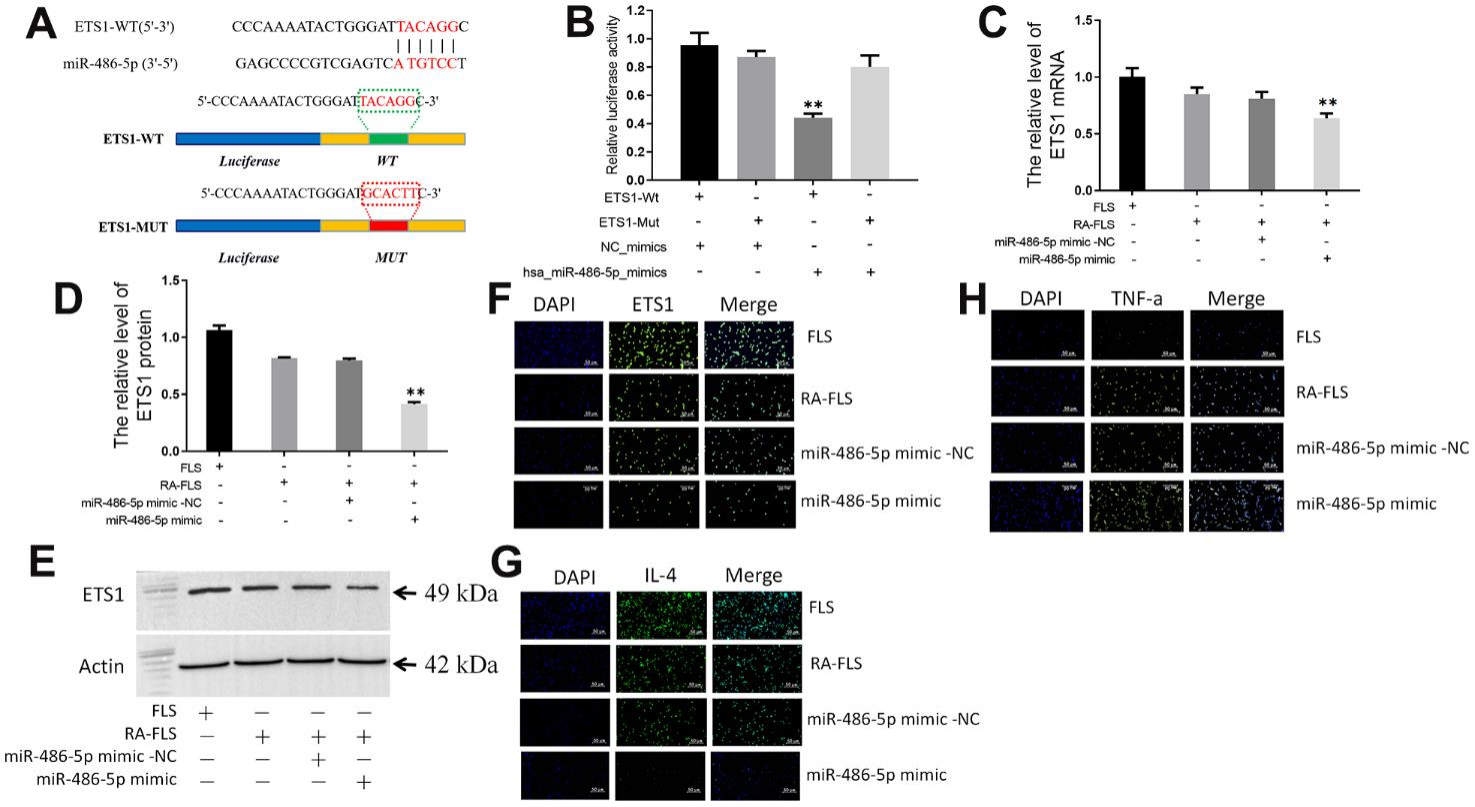
**circ_0066715 regulates the miR-486-5p/ETS1 axis.** (**A**) ETS1 binding site to miR-486-5p. (**B**) Luciferase reporter. **P < 0.01. (n=3, ANOVA with Bonferroni’s post test). (**C**) ETS1 mRNA expression in miR-486-5p overexpressing RA-FLS cells. **P < 0.01. (n=3, ANOVA with Bonferroni’s post test). (**D**, **E**) Expression of ETS1 protein in miR-486-5p overexpressed RA-FLS cells was performed by western blotting. **P < 0.01. (n=3, ANOVA with Bonferroni’s post test). (**F**) ETS1 expression in FLS cells (IF,×200). (**G**) IL-4 expression in FLS cells (IF,×200). (**H**) TNF-α expression in FLS cells (IF,×200).

By searching the miRWalk database, we found that ETS1 may be a target gene of miR-486-5p. We then mutated the sites for their binding (Figure 4A). A luciferase reporter gene assay was performed, whose results showed that miR-486-5p overexpression significantly reduced the luciferase activity of the wild-type ETS1-3`-UTR vector, but not of the mutant (Figure 4B).

Furthermore, both ETS1 mRNA and protein levels were lower in miR-486-5p-overexpressing RA-FLS cells than those in the control group (Figure 4C–4E). Immunofluorescence detection showed that overexpression of circ_0066715 significantly increased the levels of ETS1 and IL-4 in RA-FLS cells. Meanwhile, TNF-α levels were reduced. Nevertheless, the overexpression of miR-486-5p effectively abolished the above effects (Figure 4F–4H).

### circ_0066715 delays macrophage polarization *in vivo*

We established an adjuvant arthritis (AA) rat model through animal experiments and observed the *in vivo* effects of circ_0066715. As can be seen in Supplementary Figure 3A–3D, the joint morphology, arthritis index, and toe swelling of the rats in the circ_0066715 overexpression group were significantly lower than those in the control group. Cytokines secreted by M1-type macrophages were decreased in the peripheral blood expressing circ_0066715. M2-type cytokines were elevated (Supplementary Figure 3E–3G). Likewise, we found that ETS1 mRNA and protein levels were significantly higher in circ_0066715-overexpressed synovium than in the controls (Supplementary Figure 3H). These *in vivo* data are consistent with our *in vitro* findings.

### Effects of ETS1 m6A methylation modification on macrophage polarization

**Figure 5:**
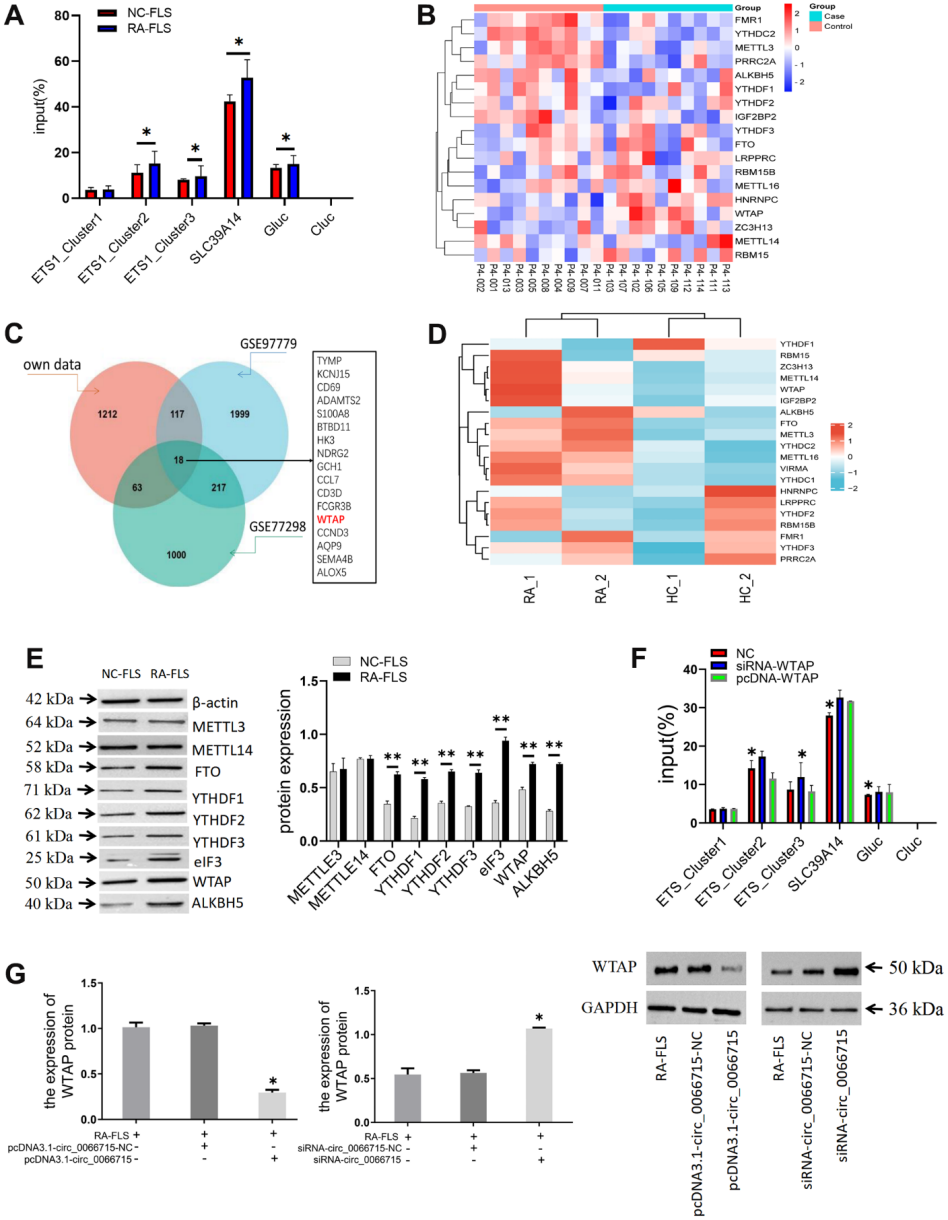
**The effect of ETS1 m6A methylation modification on macrophage polarization.** (**A**) Analysis of m6A methylation modification sites and ETS1-m6A-qPCR.*P < 0.05. (n=3, paired t-test). (**B**) Heatmap of genes associated with m6A RNA methylation. (**C**) Venn diagram of methylation-related genes in the dataset. (**D**) Analysis of the mRNA expression of the main modification enzymes of m6A RNA methylation. (**E**) m6A methylase protein expression in co-cultured cells by western blotting. **P < 0.01, vs NC-FLS. (n=3, paired t-test). (**F**) ETS1 methylation expression after WTAP intervention. **P < 0.01, *P < 0.05, vs NC. (n=3, paired t-test). (**G**) The effect of circ_0066715 on WTAP. *P<0.05. (n=3, ANOVA with Bonferroni’s post test).

SLC39A14 was used as positive control, unmodified control RNA (CLuc) as negative control, and m6A modified control RNA (GLuc) as a reference. We predicted that three m6A methylation modification sites (ETS1_Cluster1, ETS1_Cluster2, and ETS1_Cluster3) might be present on ETS1mRNA. Therefore, specific primers were designed for these three sites and detected in the subsequent experiment. No significant differences were established from the positive and negative control treatments in the m6A-qPCR results of Cluster1 in NC-FLS and RA-FLS, but there were differences between Cluster2 and Cluster3. Therefore, two m6A methylation modification sites exist in the ETS1 mRNA (Figure 5A). Further, we conducted differentially expressed gene (DEG) dataset analysis to screen for methylated genes (Figure 5B–5D).

The differentially expressed genes in the dataset were next validated. The detection of m6A methylase showed that WTAP, YTHDF1-3, FTO, ALKBH5, and eIF3 were differentially expressed. No difference was found in the expression of METTL3 and METTL14 (Figure 5E). Then, the overlapping gene WTAP was derived by constructing the methylation-related genes in the dataset. We chose WTAP in our follow-up studies and speculated that WTAP might affect the ETS1 methylation process in RA. We intervene with WTAP. We found that the m6A methylation level of ETS1 was increased by the knockout of WTAP. On the other hand, WTAP overexpression decreased the methylation level of ETS1 m6A. There is thus a direct relationship between WTAP and m6A methylation of ETS1 (Figure 5F). We then aimed to verify whether circ_0066715 affects the expression of the downstream transcriptional gene ETS1 by acting on WTAP. We found that the expression of WTAP decreased or increased after overexpression or interference of circ_0066715. This indicated that circ_0066715 could affect the WTAP methylation process (Figure 5G).

## DISCUSSION

The functions of macrophages are highly heterogeneous and plastic. Macrophages can be classified into M1 and M2 types [7, 20, 21]. M1 macrophages are stimulated and activated mainly by GM-CSF, LPS, etc., and can secrete a variety of pro-inflammatory cytokines (IL-1beta, IL-23, and TNF-α, etc.), CD80, CD86, and other molecules that can promote inflammation [10, 22, 23]. M2 macrophages are stimulated and activated predominantly by M-CSF, IL-4, etc., followed by the overexpression of anti-inflammatory cytokines, such as IL-10, vascular endothelial growth factor, matrix metalloproteinase 1, and CD206 and CD163 molecules. Inhibits the proliferation, activation and anti-inflammatory effects of T cells [4, 24, 25]. Synovial macrophages in RA patients have an M1 pro-inflammatory polarized phenotype [4]. In this study, we found that the expression of M1 macrophage marker CD14+CD86+ in RA patients was higher than that in with normal subjects. Meanwhile, the expression of the cytokines IL-1R1, IFN-γ, and iNOS2 secreted by M1 macrophages also increased. Similarly, abnormally expressed M1 macrophages were also found in our animal experiments. The abnormalities of these indicators suggest that RA inflammation is dominated by M1 macrophages. Therefore, it can be speculated from the results of this study that peripheral blood mononuclear cells of RA patients tend to be polarized by M1-type macrophages. Correlation analysis also showed that M1 macrophage markers and cytokines were associated with RA disease. The pro-inflammatory cytokines IL-1R1, IFN-γ, and iNOS2 secreted by these cells not only aggravate the existing inflammatory response, but also activate the FLS around the articular cartilage to secrete a variety of proteases and collagenases that split collagen and hyaluronic acid, resulting in joint tissue destruction, aggravating RA disease.

miR-486-5p is involved in a variety of inflammatory processes by regulating the occurrence of inflammatory responses [26, 27]. miR-486 is an important inflammatory regulatory molecule that is overexpressed in the FLS and peripheral blood lymphocytes of RA patients [28]. miR-486 levels correlate with arthritis activity, and inflammatory mediators were found to induce miR-486 level elevation in normal human fibroblast macrophages and monocytes [29]. Additionally, miR-486 was established to be associated with the progression of RA inflammation [30]. The present study found that the overexpression of miR-486-5p significantly reduced the luciferase activity of the wild-type ETS1 vector, but not of the mutant. Hence, miR-486 targets the expression of ETS1 and affects the expression of downstream transcription factors. Further investigations showed that ETS1 mRNA and protein were decreased in RA-FLS cells with high miR-486-5p expression; while TNF-α was decreased, IL-4 was increased. This indicated that miR-486 inhibited the expression of ETS1, which in turn promoted the synovial and joint inflammatory responses, leading to the occurrence of RA synovial inflammation and bone destruction.

circRNAs are closed circular non-coding RNAs formed by covalent bonds [31–33], which play an important role in the pathogenesis of RA [34, 35]. Our previous studies have shown that abnormally expressed circRNAs exist in the blood and synovial tissue of RA patients. circ_0066715 may be involved in the regulation of the level of inflammation *in vivo*. In this study, we found that RA disease activity was closely related to the low expression of circ_0066715. These findings suggested that circ_0066715 could regulate inflammatory polarization in macrophages and synoviocytes and is thus involved in RA synovial inflammatory response. Here, we established that the overexpression of circ_0066715 significantly reduced FLS cell viability and number. This indicated that circ_0066715 inhibits FLS proliferation. Notably, a co-expression relationship exists between circRNAs and miRNAs. After its transcription, circRNAs cooperate with miRNAs to participate in the regulation of RA [36, 37]. There is also a regulatory relationship between circ_0066715 and miR-486 in this study. circ_0066715 sequesters miR-486 from the target gene mRNA (ETS1) through a “sponge” effect, thereby inhibiting the regulatory effect of miR-486. Further, circ_0066715 recruits the pre-miR 486 splicing factor to gene transcription sites in the nucleus. Finally, it regulates the expression of the target gene ETS1. In addition, circ_0066715 inhibits cell viability by downregulating miR-486 expression. This in turn promotes the expression of the target gene ETS1 and inhibits the polarization of M1 macrophages, reducing RA synovial inflammatory response. Conversely, the regulatory function of circ_0066715 is weakened and it could not exert the “sponge-like” adsorption effect of endogenous miRNA and thus promotes synovial inflammation exacerbation.

m6A methylation modifications are necessary for the immune response process [38, 39]. Enzymes, such as methyltransferases, demethylases, and binding proteins, are involved in m6A regulation [40, 41]. m6A methylation modification is also crucial in RA development [14, 42, 43]. m6A methylation is an abundant internal modification on RNA [44–46]. The binding protein can recognize the m6A methylation site of mRNA, affecting the abundance and function of mRNAs with m6A modification sites. mRNA expression in RA is also affected [13, 38, 47]. In this study we constructed a methylation-related gene dataset. The overlapping genes WTAP and FTO were derived, and FTO was found to be a demethylated gene. In addition to the role of methyltransferase, WTAP also recruits METTL3 and METTL14. Consider the functions of these two genes, in combination with our validation results, we chose to further examine WTAP. Interestingly, here we found that ETS1 m6A methylation levels were elevated after WTAP was knocked out. In contrast, the overexpression of WTAP reduced the methylation level of ETS1 m6A. Meanwhile, the expression of WTAP decreased after the overexpression of circ_0066715. Nevertheless, the interference with circ_0066715 increased the expression of WTAP. Therefore, circ_0066715 affects the WTAP methylation process and the expression of the downstream transcriptional gene ETS1, influencing macrophage polarization in RA. The mechanism diagram is shown in Supplementary Figure 4.

In summary, this study yielded only a preliminary discovery of key genes involved in RA immune inflammation. Questions, such as what are the associations and interactions among circRNAs, miRNAs in gene co-expression and whether ceRNAs form a co-expression network profile that binds to downstream proteins affects transcription, still need to be answered. Additionally, it is still unclear how m6A methylation modification of circRNA further regulates mRNA, which ultimately affects macrophage polarization. Finding the answers to these research questions will be the purpose of our future further investigations. Therefore, we will further investigate the modification of mRNA by the m6A methylation protein of circRNA, which ultimately affects the inflammatory polarization of RA macrophages.

## MATERIALS AND METHODS

### Clinical samples

To search for differentially expressed circRNAs and miRNAs in RA, we collected six paired RA and healthy control (HC) mononuclear cells. In addition, 15 pairs of RA and HC blood samples were collected to verify circRNA and miRNA expression. We obtained written informed consent from each of the enrolled patients. This study was conducted after the approval of the hospital ethics committee (2019AH-12). All enrolled patients signed a written informed consent form. A RA synovial fibroblast (FLS) cell line (RA-FLS, Saibaikang, Shanghai, China) was purchased and cultured in DMEM medium with 10% fetal bovine serum (FBS).

### Laboratory animals

The present animal studies were approved by the Hospital Animal Ethics Review Committee (AHUCM-rats-2021022). A total number of 30 female SD rats were randomly divided into three groups (*n = 10*). The adjuvant arthritis (AA) animal model was replicated using Freund’s complete adjuvant (Sigma, Louis, MO, USA). Then, control and circ_0066715-overexpressing cells were injected subcutaneously into arthritic rats. The arthritis index and toe swelling were measured daily. All rats were sacrificed four weeks after experiment initiation. The expression of M1 and M2 cells in the peripheral blood was detected by flow cytometry, and the pathological morphology of the synovium was observed. Synovial membranes were collected for qRT-PCR and immunoblot analyses.

### Flow cytometry

The expression levels of M1 and M2 macrophage polarization markers CD86, CD163, and CD206 were detected by flow cytometry. In 100 μL of peripheral blood anticoagulated with heparin sodium, we added mouse anti-human CD14-FITC, CD86-PE-Cyanine5, CD163-APC, and CD206-PE (Biolegend, San Diego, CA, USA) to 10 μL respectively. After the sample was mixed well, it was incubated in the dark at room temperature for 20 min, followed by the addition of red blood cell lysate. Further, each sample was shaken and incubated in the dark at room temperature for 15 min, followed by flow cytometry measurements. Then, the forward scatter (FSC) was taken as the abscissa and the longitudinal scatter as the ordinate (SSC) to develop a scatter plot. The lymphocyte gate was set, and then the boundary fluorescence marker was determined. The results were analyzed with Flow Jo 7.6.1 software.

### Cell proliferation assay

RA-FLS cells were seeded on 96-well plates and cultured for 24, 48, and 72 h, followed by the addition of 10 μL of CCK-8 solution. Next, the absorbance of each well was measured after 2-h incubation at 37° C. In the cell proliferation assay, cells were seeded into 6-well plates and then cultured and stained with crystal violet. Finally, the proliferation was determined and recorded.

### Cell transfection and infection

cDNA of ETS1 was cloned into a pcDNA3.1 plasmid by homologous recombination to construct an ETS1 overexpression vector, and RA-FLS cells were transfected with Lipofectamine 2000 (Invitrogen, Carlsbad, CA, USA). In addition, siRNA targeting ETS1 was synthesized using an RNAi MAX transfection reagent (Jima, Shanghai, China) and transfected into RA-FLS cells. To stably overexpress circ_0066715, it was cloned into a pcDNA3.1 vector by homologous recombination. The recombinant plasmids were transfected into RA-FLS cells using Lipofectamine 2000 (Invitrogen, Carlsbad, CA, USA). The validity of the infection and transfection was verified by qRT-PCR.

### Luciferase reporter assay

To determine the regulatory axis of circ_0066715/miR-486-5p/ETS1, the luciferase reporter gene plasmids of circ_0066715 and ETS1 were first constructed. The modified plasmids contained the predicted binding site of miR-486-5p. Functional cells were then co-transfected with miR-486-5p mimic using Lipofectamine 2000. Further, 48 h post-transfection, the luciferase activity was measured with the Promega Dual Glo® Luciferase Assay System (Promega Corporation, Sunnyvale, CA, USA).

### RNA immunoprecipitation (RIP)

After collecting the target cells, specific magnetic beads were used to enrich the protein. Then, the enriched RNA was purified and subjected to RT-qPCR. In the RIP AGO2 antibody experiment, IgG was used as a negative control, and AGO2 was the core member of the intracellular RISC-induced silencing complex. By AGO2 RIPing, the intracellular RNA-silencing complex and the RNA-silencing complex bound in RISC were enriched. The resulting RNA was enriched for RT-qPCR analysis.

### Nucleus/cytoplasm separation

Isolation of nuclear/cytoplasmic fractions was performed using a nuclear and cytoplasmic extraction kit (Norgen Biotek, Thorold, ON, Canada). GAPDH and U6 were utilized as reference controls for the cytoplasm and nucleus, respectively.

### RNA fluorescence *in situ* hybridization (FISH)

RA-FLS cells were incubated with 4% formaldehyde for 15 min at room temperature. Then, the cells were incubated in 70% ethanol for 1 h at 4° C, washed with PBS at room temperature, and hydrogenated for 12 h at the circ_0066715-binding site using labeled probes in a humidified chamber at 37° C. After rinsing with SSC for 30 min at room temperature, the cells were counterstained with DAPI, and images were acquired using a microscope.

### RNA pull down

Biotin-labeled probes targeting the circ_0066715 junction site were synthesized and used following the manufacturer’s instructions provided with the RNA pull-down kit (Saicheng, Guangzhou, China). Cell lysates were prepared with IP lysis buffer. The beads were washed twice with 20 mM Tris (pH = 7.5) and resuspended with the same volume of 1× RNA capture buffer. Then 50 pmol of labeled probe was added to the beads and stirred for 30 min at room temperature. The supernatant was discarded and washed twice. RNA-binding buffer was added to the beads, and the supernatant was discarded and incubated at 4° C with rotation for 1 h. After washing, circ_0066715-enriched miRNAs were eluted and extracted with TRIzol reagent, followed by qRT-PCR detection.

### m6A methyltransferase activity assay

A universal methyltransferase activity assay kit (Aimeijie, Wuhan, China) was used. m6A methyltransferase activity was determined according to the manufacturer’s instructions. Samples were mixed with 100 ng RNAs and negative or positive controls, or methylase at various dilutions. The fluorescence signal was measured at 380eX/520eM. The m6A methyltransferase activity of FLS cells was finally observed.

### Actinomycin D transcription inhibition assay

WTAP knockdown or overexpression (pcDNA3.1) vectors were constructed by homologous recombination, which vectors were consistent with the previous description. To verify the effect of WTAP knockdown/overexpression on ETS1 stability, 2 μg/mL actinomycin D was added to the WTAP knockdown/overexpressing cell line to inhibit the transcriptional process. Cells were harvested at 8, 16, and 24 h post-treatment. After the cells were treated with actinomycin D, the content of target RNA was detected by qPCR. We determined the stability of the target RNA; ETS1 stability was detected by RT-qPCR.

### qRT-PCR analysis

Total RNA was isolated from RA peripheral blood and cells using a TRIzol kit (Life Technologies, Waltham, MA, USA), and 1 μg of RNA was used to generate cDNA with an RT kit (TaKaRa, Tokyo, Japan) following the manufacturer’s instructions. RNA quantification assays were next performed using SYBR master mix. Using GAPDH as an internal reference, the relative levels of each gene were analyzed by the 2^−ΔΔCt^ method.

### Western blot

Total protein was extracted with a protein extraction kit (Beibo, Shanghai, China and transferred from the gel to PVDF membranes (Millipore, Bedford, MA, USA) using a semi-dry method (Tianneng, Shanghai, China). Then, the membrane was blocked with blocking solution and incubated overnight at 4° C. with rabbit anti-human ETS1 (Bioss, Beijing, China), rabbit anti-human WTAP, rabbit anti-human YTHDF1, rabbit anti-human YTHDF2, rabbit anti-human YTHDF3, rabbit anti-human FTO, rabbit anti-human ALKBH5, mouse anti-human eIF3, Rabbit anti-human METTL3, and mouse anti-human METTL14 (Abcam, Biotechnology, Cambridge, UK) primary antibodies. Further, the membrane was bound to HRP and incubated with secondary antibodies (goat anti-mouse IgG, Zsbio, Beijing, China; goat anti-rabbit IgG, Zsbio, Beijing, China) at room temperature for 2 h. The intensity of protein expression was detected by ECL chemiluminescence (Thermo Corp., Waltham, MA, USA), with β-actin and GAPDH as an internal normalization control. The strip image processing software is Image J.

### Enzyme linked immunosorbent assay(ELISA)

The inflammatory cytokines of cell supernatant were detected by ELISA kits (Wuhan Jiyinmei Technology Co., Ltd, Wuhan, China). Similarly, the absorbance at 450 nm was measured by microplate reader.

### Statistical analysis

Clinical and experimental data were statistically analyzed using SPSS 23.0 software (IBM, Armonk, NY, USA). Statistical differences and graph production were assessed using Graph Prism software. For the comparison of the calculated mean values of each of the aspects, student’s unpaired, two-tailed t-tests were used and confirmed by One-way ANOVA analysis followed by a Bonferroni’s multiple comparison post-hoc tests. Comparisons between groups were performed using *t*-test. Data were pooled for Cell viability, clone rate, and circRNA/miRNA/mRNA expression, thus SD-values are shown, percent apoptosis, M6A methylase average values and data for the macrophage polarization markers were evaluated as mean of means, thus SEM-values indicate error. Correlation analysis was used for circ_0066715, macrophages and RA disease indicators. *P* < 0.05 was considered to indicate statistically significant differences.

### Data availability statement

The data set supporting the results of this article are included within the article and supplementary material.

## Supplementary Material

Supplementary Figures
