# Supplementary material for: Role of m6A modification and novel circ_0066715/ miR-486-5p/ ETS1 axis in rheumatoid arthritis macrophage polarization progression
Source: Aging (Albany NY). 2022 Dec 20;14(24):10009–26. doi: 10.18632/aging.204439 (PMC9831719; doi:10.18632/aging.204439)
Supplement: Supplementary Figures [file aging-14-204439-s001.pdf]

SUPPLEMENTARY MATERIALS

Supplementary Figures

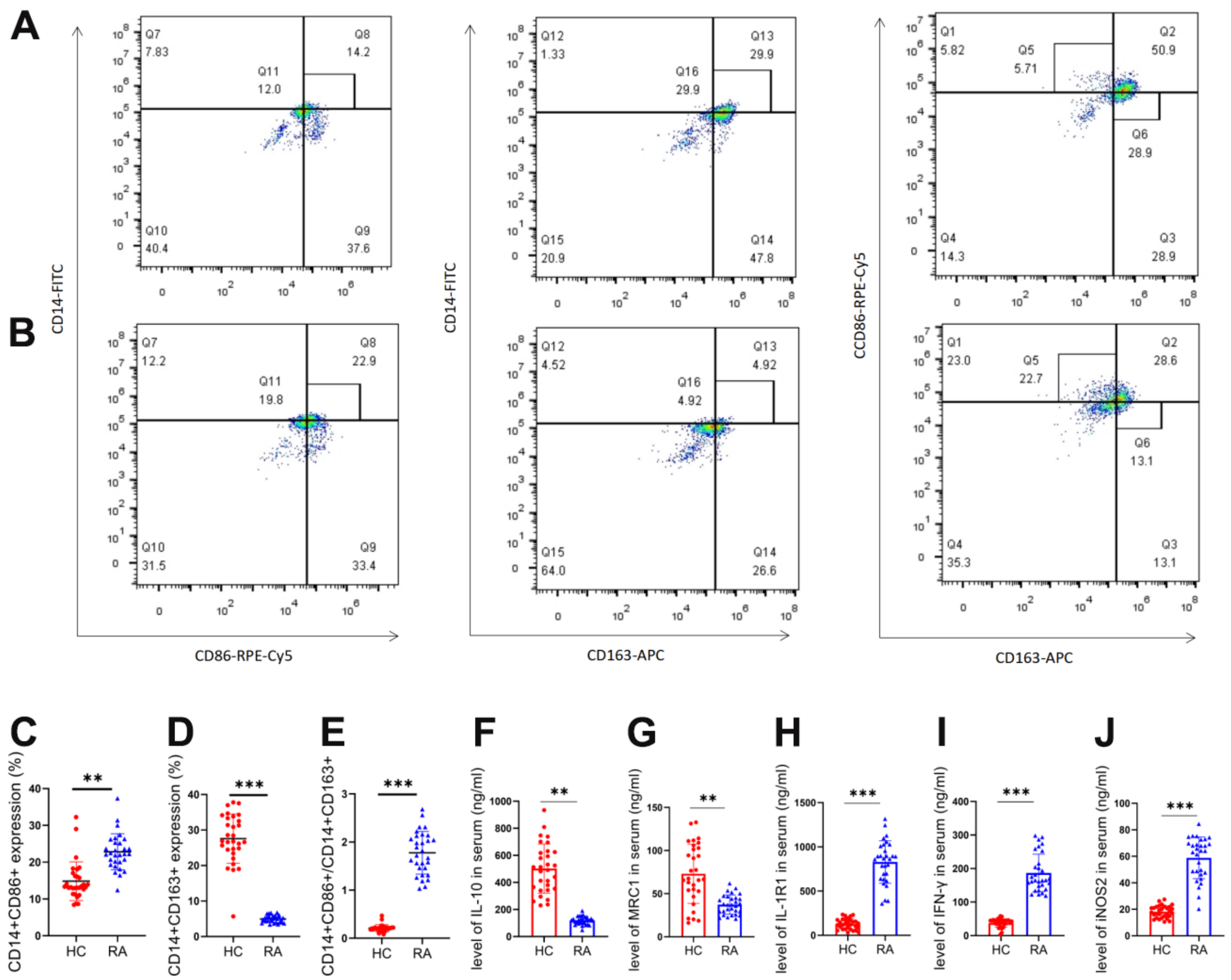

Supplementary Figure 1. Comparison of M1 and M2 macrophage markers between the two groups. (**A**) Flow cytometry of macrophages in the HC group. (**B**) Flow cytometry map of macrophages in RA group. (**C**–**F**) Comparison of HC and RA macrophage markers. (**G**–**J**) Comparison of cytokine secretion by HC and RA macrophages. ****P*<0.001, ***P*<0.01.

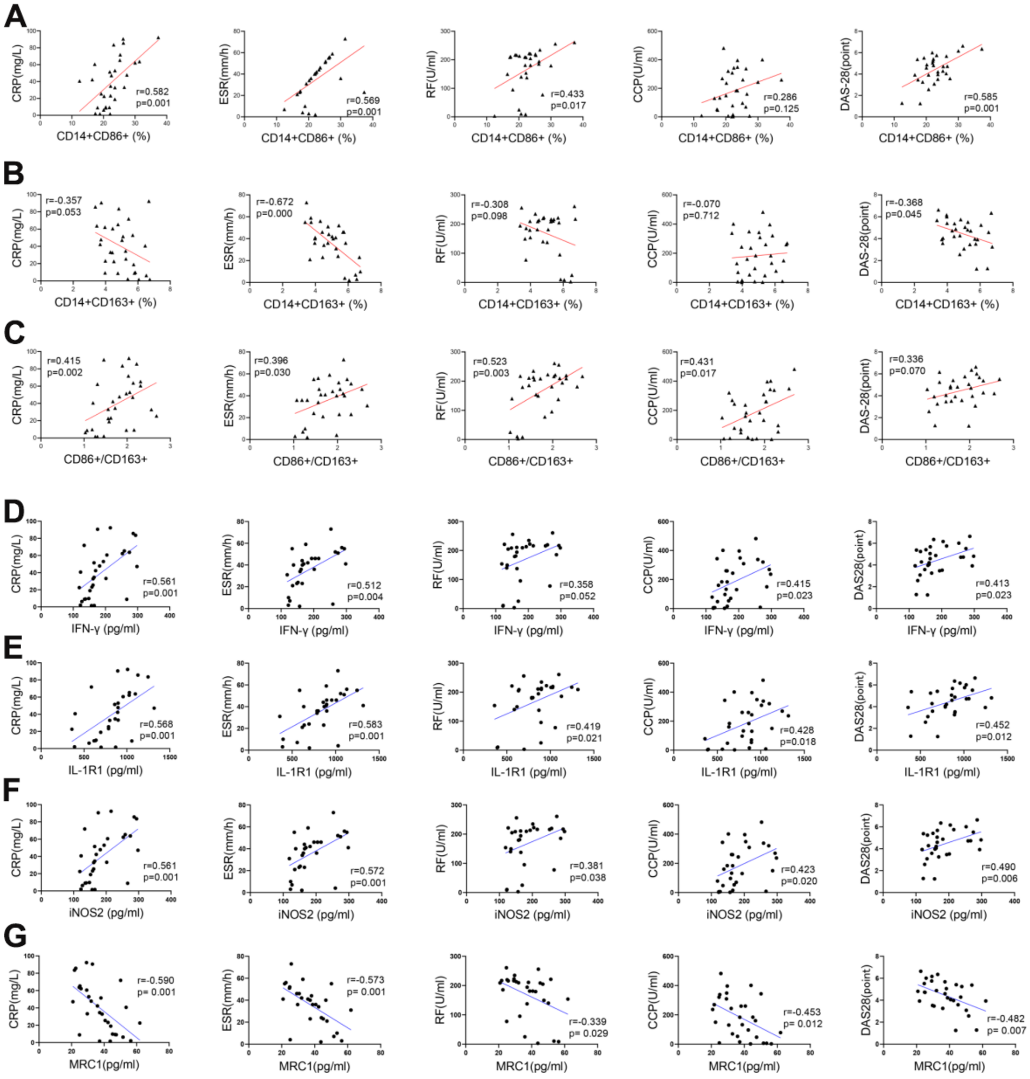

Supplementary Figure 2. Correlation analysis between M1 and M2 macrophages and clinical indicators of RA. (**A**) The correlation between CD14+CD86+ and clinical indicators of RA. (**B**) The correlation between CD14+CD163+ and clinical indicators of RA. (**C**) The correlation between CD86+/CD163+ and clinical indicators of RA. (**D**) The correlation between IFN-γ and clinical indicators of RA. (**E**) Correlation of IL-1R1 with clinical indicators of RA. (**F**) Correlation of iNOS2 with clinical indicators of RA. (**G**) Correlation of MRC1 with clinical indicators of RA.

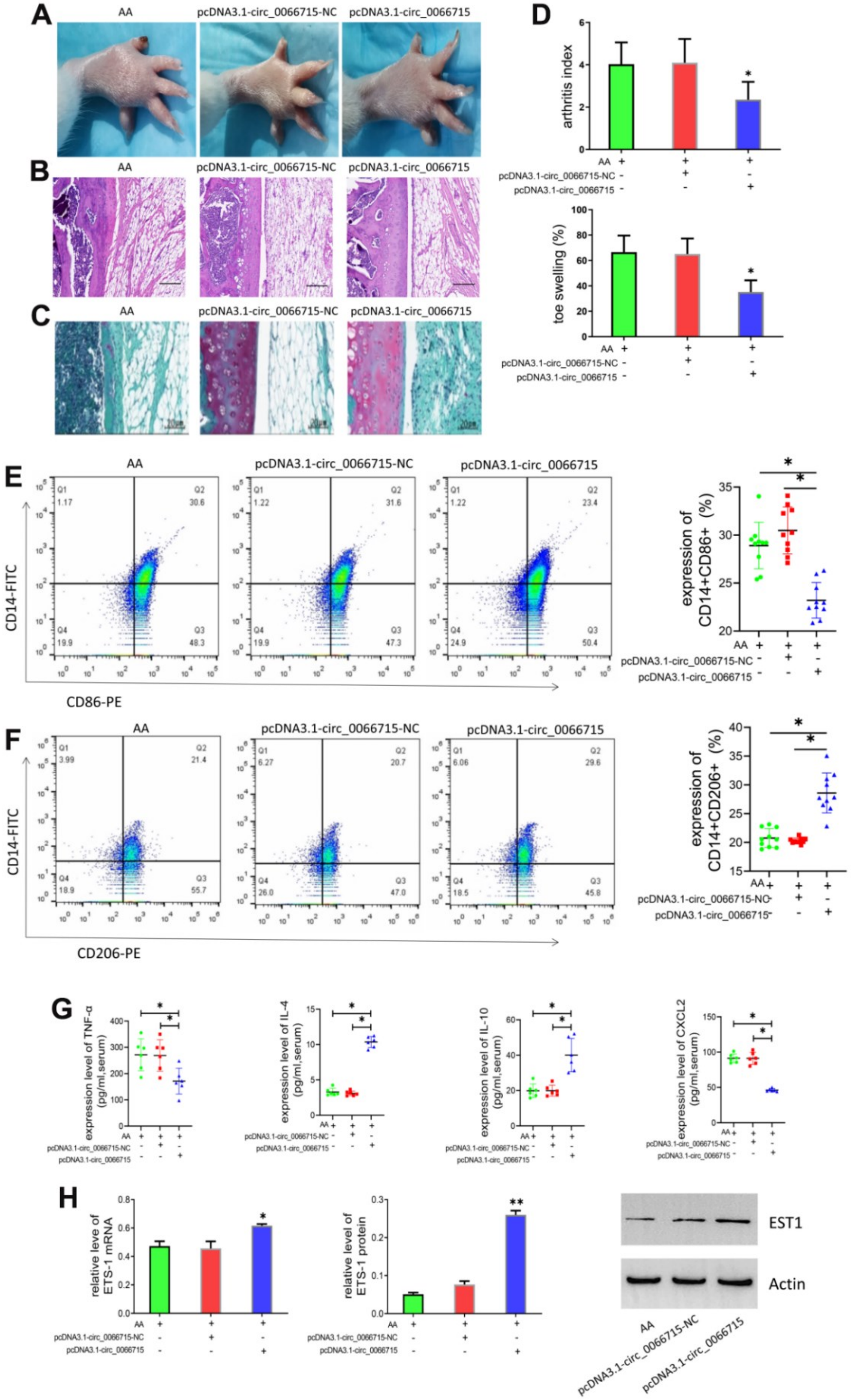

Supplementary Figure 3. circ_0066715 delays macrophage polarization *in vivo.* (**A**) Comparison of joint morphology; (**B**) Comparison of joint HE staining (×200); (**C**) Comparison of joint safranine fast green (×200); (**D**) Comparison of arthritis index and toe swelling; (**E**) M1 macrophage marker comparison; (**F**) M2 macrophage marker comparison; (**G**) Comparison of cytokine secretion by macrophages; (**H**) ETS1 changes after circ_0066715 overexpression. ** $P < 0.01$, * $P < 0.05$.

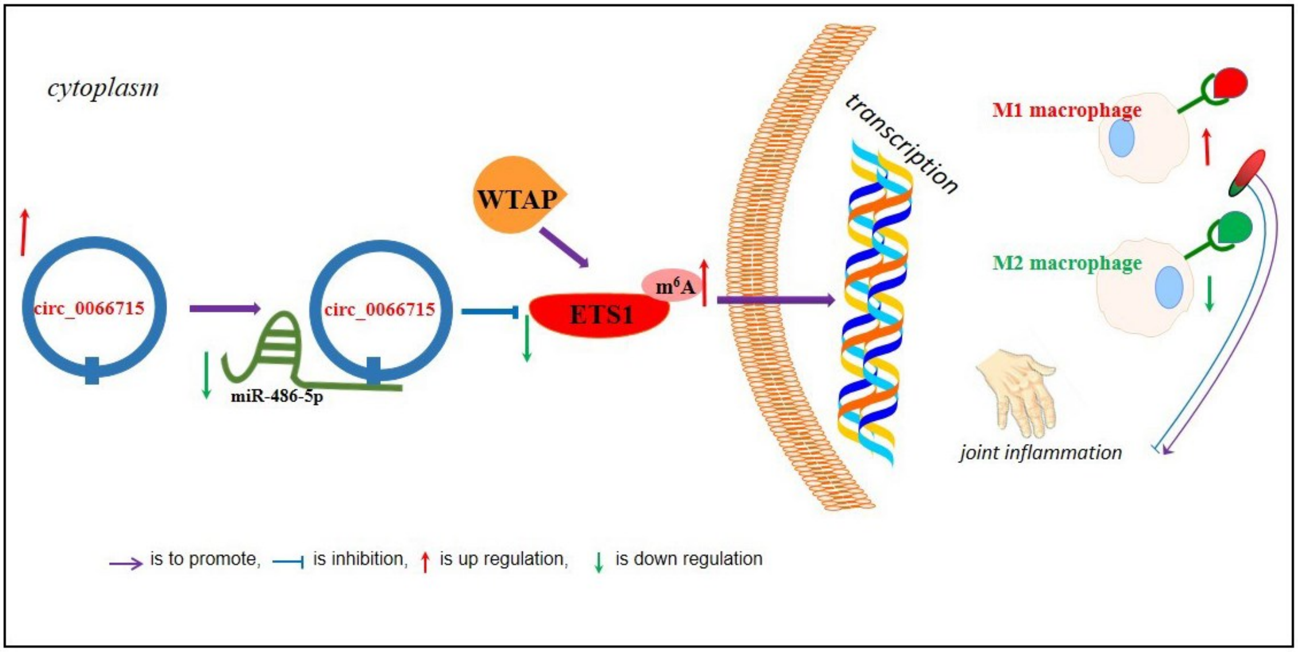

Supplementary Figure 4. Mechanistic process of ceRNA regulatory axis and m6A methylation modification involved in RA macrophage polarization.
